# Recent advances in applications of artificial intelligence-assisted Raman spectroscopy in diagnosis of cancers

**DOI:** 10.3389/fmolb.2025.1690063

**Published:** 2025-12-11

**Authors:** Xinran Zhu, Yanfu Zhao, Chunfang Zan, He Ma, Jingxin Liu

**Affiliations:** 1 Department of Radiotherapy, China-Japan Union Hospital of Jilin University, Changchun, China; 2 Department of Radiology, China-Japan Union Hospital of Jilin University, Changchun, China; 3 Collaborative Innovation Center for Molecular Imaging of Precision Medicine, Shanxi Medical University, Taiyuan, China; 4 Department of Anesthesiology, The Second Hospital of Jilin University, Changchun, China

**Keywords:** Raman spectroscopy, artificial intelligence, cancers, non-cancer diseases, diagnosis

## Abstract

Cancer remains one of the leading causes of death worldwide. Among various diagnostic approaches, Raman spectroscopy (RS) has emerged as an advanced detection technology with the potential to distinguish cancerous tissues from normal ones. Notably, RS has been verified to show improved sensitivity, specificity, and accuracy for cancer diagnosis compared to conventional techniques. Recently, artificial intelligence (AI), developed to emulate human capabilities, has gained enough popularity and showcased its strength in learning high-level representations and recognizing complex patterns with remarkable efficiency. In this context, AI-assisted RS has been applied to the classification and prediction of cancer cells, achieving a higher accuracy of ∼90% in correct predictions from a single spectrum. However, there has been no comprehensive review about the use of AI-assisted RS in distinguishing different types of cancer cells. Although AI-assisted RS has been widely utilized by researchers and clinicians over the past a few years to diagnose various cancers, including gastrointestinal, head and neck, cervical, and endocrine-related cancers, an in-depth investigation has yet to be conducted. This review aims to provide a narrative overview of the latest applications of AI-assisted RS in cancer diagnosis, summarize the key findings and benefits, discuss the associated challenges in different types of cancers, and present additional studies on AI-assisted RS in non-cancer diseases, such as fungal infections. Through this review, we hope to enhance researchers’ understanding of the potential value of AI-assisted RS in both cancer and non-cancer diseases, presenting a new diagnostic approach for clinical management, optimizing diagnostic efficacy, and ultimately improving patient survival outcomes.

## 1 Introduction

Cancer, classified as a malignant tumor, represents a significant global public health issue and poses a serious threat to human life and wellbeing (Sung et al., 2021; Duan et al., 2024). According to the World Health Organization (WHO), nearly 10 million deaths, or approximately one-sixth of total deaths, were attributed to cancer in 2020 (Castro-Muñoz et al., 2023). Early diagnosis, real-time monitoring, and personalized treatment are three critical factors that indeed improve the survival rate and extend the lifespan of cancer patients. Among current methods for early cancer diagnosis, Raman spectroscopy (RS) has emerged as a powerful spectroscopic technique with promising applications in clinical oncology due to its label-free, non-invasive, non-destructive, and rapid characteristics (Krzemińska et al., 2025; Wu et al., 2022). Notably, RS has a unique advantage in differentiating oncological samples from non-pathological ones. This capability not only aids in distinguishing malignant lesions from benign ones with high sensitivity and specificity but also helps identify specific types of cancers (Jain et al., 2024; Hanna et al., 2024; Liu X. et al., 2024). Furthermore, RS can detect molecular markers related to malignant transformations, such as extracellular vesicles, making it a valuable diagnostic tool for the early detection of precancerous and cancerous lesions *in vivo* (Das et al., 2023; Li et al., 2022). To date, RS has gained increasing recognition for its clinical utility in differentiating and diagnosing cancer cells in various cancers, including brain tumors (Doran et al., 2022), oral cancer (Li X. et al., 2023; Li et al., 2024), gastric cancer (Du et al., 2023), and skin cancer (Wang et al., 2024; Delrue et al., 2023). This advancement enhances intraoperative decision-making by providing rapid and reliable identification of invasive cancer, thus minimizing residual tumor volume and ultimately improving patient survival outcomes.

Despite the wide applications of RS in cancer diagnostics, several challenges persist (Albagieh et al., 2025; Krynicka et al., 2025). For instance, its effectiveness is limited by the complexity in analyzing Raman data to identify vibrational fingerprints (Blake et al., 2022). Moreover, distinguishing an individual with a specific disease from others who may have multiple diseases through RS proves to be challenging (Hu et al., 2025). To address this issue, the integration of artificial intelligence (AI) systems could greatly enhance the detection of statistically significant differences in spectral data, thereby improving prognostic and diagnostic stages through precise and comprehensive cellular analysis (Chadokiya et al., 2025; Bi et al., 2024a). AI is the field of science and engineering dedicated to creating computer systems that simulate human capabilities in cognition, perception, and decision-making, yielding substantial benefits in research, healthcare, and industry (Basubrin, 2025; Chen et al., 2025; Smits Serena et al., 2025). Particularly, AI is well-suited for processing Raman images, as these images contain rich information. Not only can AI efficiently handle large-scale data analysis, but it is also particularly adept at processing two-dimensional images (Shetty et al., 2025; Fernandes et al., 2025). Furthermore, advanced AI models, such as convolutional neural networks and residual networks, have demonstrated improved capabilities in analyzing and classifying cancer samples (Qi et al., 2023).

It is well established that significant efforts have been made to employ AI methods for identifying and analyzing characteristic spectral patterns used in cell classification, thereby achieving high diagnostic accuracy (Ghete et al., 2024; Zare Harofte et al., 2022). Notably, scientific evidence supports the application of RS in conjunction with machine learning approaches to classify various tumor cell types and differentiate between cancerous and non-cancerous cells (Zhang et al., 2022; He et al., 2022). For instance, many machine learning and RS-based techniques for distinguishing and classifying human liver cancer cells from non-cancer cells have utilized immortalized cell lines. To date, AI-assisted RS has been extensively applied in the diagnosis of multiple cancers, including gastrointestinal cancers, head and neck cancers, cervical cancer, and endocrine-related cancers. However, there has yet to be a systematic review of the use of AI-assisted RS specifically for recognizing different types of cancer cells. Of note, compared to the prior RS/cancer reviews, this narrative review has a broader cancer coverage, a special focus on the integration of RS/AI, and the latest relevant literature.

**FIGURE 1:**
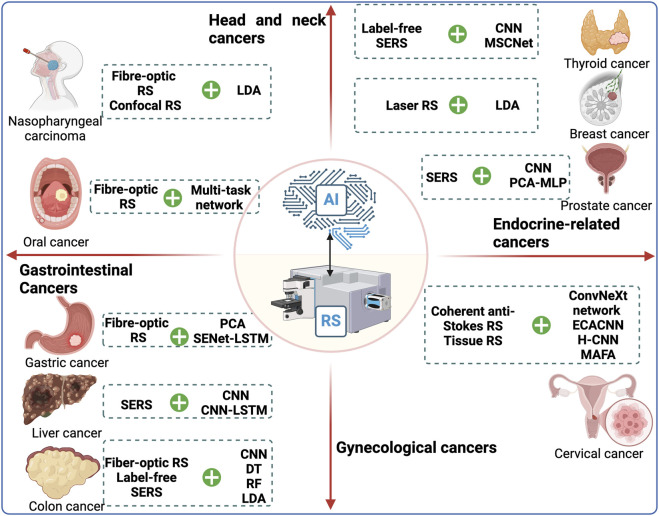
A summary of applications of combination of RS patterns and AI algorithms in diagnosis of cancers. Depicted are representative studies of various RS patterns and AI algorithms in different types of cancers. Abbreviations: AI, artificial intelligence; CNN, convolutional neural network; DRSN, deep residual shrinkage network; LDA, linear discriminant analysis; LR, logistic regression; MCNN, multi-scale convolutional neural network; PCA-RF, principal component analysis with random forest; ResNet, residual network; RS, Raman spectroscopy; SERS, surface-enhanced Raman spectroscopy.

Based on this background information, we aim to provide a comprehensive overview of the most up-to-date applications of AI-assisted RS in cancer diagnosis. We will summarize the major findings, advantages, and associated challenges of AI-assisted RS across different cancer types (see Table 1; Figure 1). Additionally, we will present studies related to the application of AI-assisted RS in non-cancer diseases, such as fungal infections, in the following sections (see Table 2). This knowledge will enhance researchers’ understanding of the potential value of AI-assisted RS in both cancer and non-cancer diseases, offering new diagnostic approaches for clinical management, optimizing diagnostic efficacy, and ultimately improving patient survival outcomes.

**TABLE 1:** Representative studies of artificial intelligence-assisted Raman spectroscopy in diagnosis of cancers.

Cancer types	RS patterns	AI algorithms	Species	Specimens	Diagnosis performance	References
Gastrointestinal cancers						
Gastric cancer	RS	Undefined	Patients	Cancer tissues	An accuracy of 89.0%, a specificity of 80.0%, a sensitivity of 89.0%	Soong et al. (2024)
	Fibre-optic RS	Undefined	Patients	Cancer tissues	Diagnostic accuracy of >85%	Ho (2022)
	RS	SENet-LSTM model	Patients with GAC	Gastric tissues	An accuracy of 96.20%	Li et al. (2023b)
	RS	PCA, machine learning	Gastric cancer and healthy patients	Serum	An accuracy of ∼95%	Guleken et al. (2023)
Liver cancer	SERS	Deep learning	Liver cancer patients and healthy volunteers	Serum	An accuracy of 99.38%, a sensitivity of 99.8%, and a specificity of 97.0%	Yang et al. (2024)
	RS	CNN-based model	Patients with primary liver cancer	Human hepatic tissue	An accuracy of 92.6%, a sensitivity and specificity of 90.8% and 94.6%	Huang et al. (2023)
	RS	CNN-LSTM model	HCC patients	Primary liver cells	An accuracy of 93%	Esposito et al. (2023)
Colorectal cancer	Fiber-optic RS	1D-CNN model	Mice; colon cancer line HCT 116	Cancer tissues	An 89.9% accuracy and 91.4% precision	Kouri et al. (2023)
	RS	CNN	Human	Colon samples	An 83% sensitivity and 45% specificity	Blake et al. (2023)
	Label-free SERS	DT, RF, and LDA	Patients with colon cancer	Serum	High diagnostic accuracy of over 90% and 100% specificity	Peng et al. (2023)
Head and neck cancers						
Nasopharyngeal carcinoma	Fiber-optic RS	Deep learning	NPC patients	NPC tissues	A 73.57% accuracy, an 89.74% sensitivity, and a 58.10% specificity	Shu et al. (2021)
	Confocal RS	LDA	NPC patients	NPC tissues; NPC cell lines	A 93.0% sensitivity, a 99.2% specificity	Xu et al. (2024)
Oral cancer	Optical fiber RS	Multi-task network models	Patients with oral cancer	Normal and cancerous tissues	An accuracy of 81.5%, a precision of 82.1%, a sensitivity of 80.2%	Li et al. (2024)
	Fiber-optic RS	Multi-task network models	Patients with oral cancer	Normal and cancerous tissues	An accuracy of 94.88%, a specificity of 99.12%, a sensitivity of 95.23%	Li et al. (2023a)
Endocrine-related cancers						
Thyroid cancer	Label-free SERS	CNN	Patients diagnosed with thyroid nodules	Thyroid FNA samples	An accuracy of 88.1%, a sensitivity of 87.8%	Gao et al. (2024)
	RS	MSCNet	Patients with thyroid and cervical lymph node malignant tumors	Serum	An accuracy of 97.95%	Song et al. (2024)
Breast cancer	RS	BP-neural networks, CNN	Patients with breast cancer	Cancerous and normal breast tissues	A 95.33% and 98.67% accuracy	Shang et al. (2020)
	Laser RS	Unsupervised k-means, stochastic nonlinear NN	Patients with breast cancer	Healthy and tumor tissue	93.2%–94.6% accuracy, 89.8%–91.8% sensitivity, and 100% specificity	Kothari et al. (2021)
	RS	Undefined	Human	Breast cancer cells	RS shows strong correlations with results of HER2 testing methodologies by IHC.	Abramczyk et al. (2024)
	RS	RF, SVM, and CNN	Mice	Breast cancer tissues	Accuracy rates were 94.47% for RF, 96.76% for SVM, and 97.58% for CNN	Zhang et al. (2024b)
	SERS	2D-CNN model	Breast cancer patients and healthy volunteers	Serum	An accuracy of 98.13%, a sensitivity of 98.65% and a specificity of 97.67%	Cheng et al. (2024)
Prostate cancer	SERS	CNN	Patients with prostate cancer or benign prostatic hyperplasia	Prostate tissues	An accuracy of 85.14%	Wang et al. (2023)
	SERS	CNN	Patients with prostate cancer	Prostate tissues	An accuracy of 99.51%, a sensitivity of 80.63%, and a specificity of 82.82%	Shao et al. (2020)
	Label-free SERS	PCA-MLP	Patients with prostate cancer	Plasma	An accuracy of 96.70% for normalized data	Ge et al. (2024)
Gynecological cancers						
Cervical cancer	Coherent anti-Stokes RS	ConvNeXt network model	Patients with cervical cancer	Cervical cancer and normal tissues	A 100% accuracy, with a loss function of 0.0927	Liu et al. (2025)
	RS	ECACNN	Patients with cervical cancer	Cervical tissues	A 94.04% accuracy	Kang et al. (2023a)
	Tissue RS	H-CNN	Patients with cervical cancer	Cervical tissues	An accuracy of 94.91%	Kang et al. (2023b)
	RS	MAFA	Patients with cervical cancer	Cervical tissues	An accuracy of 82.36%, a precision of 84.00%	Liu et al. (2023)

Abbreviations: AI, artificial intelligence; CNN, convolutional neural network; DT, decision tree; ECACNN, efficient channel attention convolutional neural network; FNA, fine-needle aspiration; GAC, gastric adenocarcinoma; HCC, hepatocellular carcinoma; H-CNN, hierarchical convolutional neural network; IHC, immunohistochemistry; LDA, linear discriminant analysis; LSTM, long-short term memory cells; MAFA, a multi-level SENet, attention mechanism feature fusion architecture; MLP, multi-layer perceptron; MSCNet, multi-modal separation cross-fusion network; NN, neural networks; NPC, nasopharyngeal carcinoma; PCA, principal component analysis; RF, random forest; RS, Raman spectroscopy; SERS, surface-enhanced Raman spectroscopy; SVM, support vector machine.

**TABLE 2:** Representative studies of artificial intelligence-assisted Raman spectroscopy in diagnosis of non-cancer diseases.

Disease types	RS patterns	AI algorithms	Species	Specimens	Diagnosis performance	References
Infections						
Fungal infection	Single-cell RS	LDA, SVM, kNN, LR	Fungi and bacteria	Clinical isolates for fungi and bacteria	An accuracy of 100%	Xu et al. (2023)
Virus co-infections	Label-free SERS	MultiplexCR	Patients	Virus mixture specimens	An accuracy of 98.6%	Yang et al. (2025b)
Inflammatory diseases						
Skin inflammation	Near-infrared RS	Multilayer perceptron algorithm	Mouse	Ears	An accuracy of 93.1%	Kanemura et al. (2022)
Atherosclerosis	SERS	PCA-PLS-DA	Apoe^−/−^ mice	Single-drop blood samples	An accuracy of 97.5%	Lee et al. (2023)
Other diseases						
Celiac disease	RS	CNN, MCNN, ResNet, DRSN	Healthy control and patients with celiac disease	Plasma	An accuracy of 86.67% for CNN, 90.76% for MCNN, 86.67% for ResNet, 95.00% for DRSN	Shi et al. (2024)
Membranous nephropathy	RS	ResNet, AlexNet and GoogleNet	Patients with membranous nephropathy	Serum, urine	An accuracy of 100% for the classification of serum data	Zhang et al. (2023b)
Radiation-induced lung injury	RS	GBR-NMF-RF, PCA-RF, CNN, LR	C3H/HeJ, C57BL/6J mice	Lung tissues	An accuracy of 95% for GBR-NMF-RF	Wiebe et al. (2024)

Abbreviations: AI, artificial intelligence; CNN, convolutional neural network; DRSN, deep residual shrinkage network; GBR-NMF-RF, group and basis restricted non-negative matrix factorization, classified with random forest; kNN, k-nearest neighbor; LDA, linear discriminant analysis; LR, logistic regression; MCNN, multi-scale convolutional neural network; PCA-RF, principal component analysis with random forest; ResNet, residual network; RS, raman spectroscopy; SERS, surface-enhanced Raman spectroscopy; SVM, support vector machine.

## 2 Incremental value of artificial intelligence for Raman spectroscopy in diagnosis of cancers and non-cancer diseases

In fact, strong background interference and a low signal-to-noise ratio, both stemming from the complex biological environment, pose significant challenges for the *in vivo* application of RS in cancer diagnosis (Bi et al., 2024b; Ru et al., 2023). Additionally, a substantial amount of Raman spectral data is required to enhance the differentiation between cancerous and normal tissues. The integration of machine learning with RS can significantly improve efficiency, providing a rapid and accurate approach for medical diagnosis (Ralbovsky and Lednev, 2020; Wang Y. et al., 2025).

The integration of artificial intelligence (AI) facilitates automated spectral analysis, enabling the rapid and precise detection of clinical samples, including serum and cancer tissues. This advancement provides a transformative solution for real-time diagnostics and significantly enhances personalized treatment strategies in the management of both cancers and non-cancer diseases (Wei et al., 2025; Nie et al., 2025). Lin and colleagues conducted a large-scale case-control study, presenting a highly effective serum surface-enhanced Raman spectroscopy (SERS)-based approach for multi-cancer early detection, which may provide new insights into cancer screening (Lin et al., 2025). The application of AI and machine learning in RS data analysis has revolutionized our approach to real-time data interpretation, particularly in single-cell and multi-omics studies (Voros et al., 2023). Despite the significant advancements driven by AI in SERS and related applications, unresolved challenges remain in various areas, including sample preparation, data acquisition, and utility (Bell et al., 2020; Lin et al., 2023; Zhou et al., 2020).

Multi-layered machine learning networks, including convolutional neural networks (CNNs), which extract high-level features from input data, have been applied to analyze Raman spectra (Qian et al., 2022; Wang et al., 2023; Yang et al., 2024). These deep learning/transformers demonstrate significant potential for integrating diverse data types, enabling researchers to characterize multiple cellular processes simultaneously (Zitu et al., 2024; Sorin et al., 2024). Furthermore, the development of semi-supervised and unsupervised machine learning models could reveal new opportunities beyond current applications (Pantanowitz et al., 2025; Abbas et al., 2024). Such advanced models may uncover hidden correlations across various omics datasets, thereby facilitating innovative hypothesis testing, drug discovery, and personalized therapeutic approaches (Zhang et al., 2024a; Sanches et al., 2024).

## 3 Applications of artificial intelligence-aided Raman spectroscopy in diagnosis of gastrointestinal cancers

Gastrointestinal cancers, which encompass malignancies affecting the digestive system—specifically the esophagus, stomach, liver, pancreas, and colorectum—rank among the most common and lethal cancers worldwide (Mishra et al., 2024; Barau et al., 2025). Enhancing the early diagnosis of gastrointestinal cancers is a pivotal step toward reducing their mortality rates (Ji et al., 2025; Mannino et al., 2025). The advent of precision medicine and the development of new technologies, such as AI, have contributed to lower mortality rates for gastrointestinal malignancies, highlighting the essential role of early detection methods in improving survival rates (Akbari et al., 2024; Nagaraju et al., 2025). Kralova and colleagues implemented an innovative approach that combines Raman spectroscopy (RS) with its conformation-sensitive polarized variant, Raman optical activity, to detect disease-specific alterations in the biomolecular structure and composition of blood plasma. This approach analyzed blood plasma samples from patients suffering from three distinct types of gastrointestinal cancer—hepatocellular, colorectal, and pancreatic—ultimately achieving an overall accuracy of 76% (Kralova et al., 2024). Furthermore, we have reviewed recent studies on AI-assisted RS for diagnosing these three types of gastrointestinal cancers: gastric cancer, liver cancer, and colorectal cancer.

### 3.1 Gastric cancer

Gastric cancer is the fifth most common cancer globally and the third leading cause of cancer-related deaths, affecting approximately one million individuals each year (Lordick et al., 2024). The Ho group has developed an endoscopic Raman fiber-optic probe that can be introduced into the gastrointestinal tract via the working channel of any endoscope for Raman measurements. Notably, they have integrated the endoscopic RS system with an AI component that classifies normal gastric tissue, gastric intestinal metaplasia, gastric dysplasia, and gastric cancer, achieving a diagnostic accuracy exceeding 85% (Ho, 2022). A year later, Li et al. developed a SENet-LSTM model for the automated classification of cancerous gastric mucosa versus normal gastric mucosa, achieving an accuracy of 96.20% with a sensitivity of 96.48% and a specificity of 95.90%. The loss is 0.208, and the AUC value is 0.99 (Li C. et al., 2023). Guleken and colleagues analyzed the dynamics of Raman spectra in gastric cancer and healthy patients, demonstrating that vibrations at 1,302 and 1,306 cm^−1^ are characteristic of cancer patients. Furthermore, the employed machine learning techniques reached a classification accuracy of over 95%, with an area under the receiver operating characteristic curve (AUROC) of 0.98, utilizing Deep Neural Networks and the XGBoost algorithm (Guleken et al., 2023). In terms of the endoscopic diagnosis of gastric neoplasia, Soong et al. compared the RS-based AI system (SPECTRA) to high-definition white light endoscopy (HD-WLE). SPECTRA achieved an overall sensitivity, specificity, and accuracy of 100% (95%CI [40%–100%]), 80% (95%CI [28%–99%]), and 89.0% (95%CI [52%–100%]) by patient, and 100% (95%CI [59%–100%]), 80% (95%CI [28%–99%]), and 92% [95%CI [62%–100%]] by lesions (Soong et al., 2024). These results illustrate that SPECTRA performs comparably to HD-WLE, indicating its potential as a valuable adjunct for less experienced endoscopists to achieve accurate and real-time diagnoses of gastric lesions (Soong et al., 2024).

In summary, these studies demonstrate that RS offers several advantages over traditional advanced imaging technologies. First, unlike confocal laser endomicroscopy (CLE), RS does not require a contrast agent for spectral acquisition (Sharma et al., 2016). Second, it does not depend on morphological information for the annotation, training, and validation of the AI system. Third, the computational AI system does not require morphological information for analysis during the procedure. Finally, as the Raman probe interrogates the target tissue through point contact, it enables precise targeting, often referred to as optical biopsy (Wood et al., 2023; Fedorov Kukk et al., 2022). These unique advantages allow the Raman-AI system to provide real-time and less operator-dependent diagnoses of gastric tissues during endoscopic examinations.

### 3.2 Liver cancer

Liver cancer ranked as the seventh most prevalent cancer and the third leading cause of cancer-related deaths worldwide in 2022 (Bray et al., 2024). Accurate and timely diagnosis is essential for the effective treatment of liver cancer and for improving survival rates (Chen et al., 2023). Current studies on liver cancer utilizing RS have predominantly focused on analyzing blood samples, with only a limited number of studies targeting human tissue (Ou et al., 2024; Yu et al., 2018). To address this gap, Huang et al. differentiated hepatic carcinoma tissues from adjacent non-tumor tissues in a rapid, non-disruptive, and label-free manner by employing RS combined with deep learning, validated by tissue metabolomics. Notably, the Raman signal derived from liver tissue blocks was sufficiently strong to be detected for diagnostic purposes in their study (Huang et al., 2023). Additionally, Esposito and colleagues evaluated primary human liver cancer cells using AI-assisted RS, demonstrating its effectiveness in tumor cell classification and prediction, achieving an accuracy of nearly 90% for correct predictions based on a single spectrum (Esposito et al., 2023). Importantly, the advantageous effects of AI-assisted RS in identifying primary human liver cancer cells have significant potential clinical applications, as evidenced by *in vitro* cytological studies that provide rapid cell analysis and *ex vivo* studies of resected tissues (Huang et al., 2023; Vardaki et al., 2023).

Yang et al. employed SERS data derived from serum samples collected from both liver cancer patients and healthy volunteers to develop and evaluate their classification model. This method, which integrates SERS, wavelet transform, and deep learning (combining Morl wavelet with Efficientnetv2), demonstrated outstanding performance, achieving an accuracy of 99.38%, a sensitivity of 99.8%, and a specificity of 97.0%. The Morl wavelet performs exceptionally well in all networks, with AUC-ROC values close to 1 (Yang et al., 2024). In comparison to traditional machine learning algorithms such as partial least squares discriminant analysis (PLS-DA), random forest, and XGBoost, the deep learning approach exhibits superior computational efficiency, particularly in accurately classifying tissues of various pathological types and effectively addressing imbalanced data (Kagawa et al., 2025; Usuzaki et al., 2024). Collectively, the integration of wavelet transform and deep learning presents considerable potential for liver cancer detection and shows promise for applications in the identification of other types of cancer.

### 3.3 Colorectal cancer

Colorectal cancer is a prevalent malignant tumor of the digestive system that arises in the colorectum, significantly impacting public health (Anbari and Ghanadi, 2025; Muradi Muhar et al., 2025). Various *ex vivo* studies have investigated the ability of RS to distinguish between cancerous and non-cancerous samples while simultaneously providing molecular information of colorectal tissue with high specificity and sensitivity (Karnachoriti et al., 2023; Synytsya et al., 2023). Kouri et al. utilized a transfer learning model based on a one-dimensional convolutional neural network (1D-CNN) to analyze Raman spectra data, assessing classification accuracy in live animals, achieving an accuracy of 89.9%, a precision of 91.4% and F1-Score of 92.1% (Kouri et al., 2023). In a similar vein, Blake and colleagues applied the CNN model and achieved 83% sensitivity, 45% specificity and the best AUC-ROC at 0.75, compared to principal component analysis-linear discriminant analysis (PCA-LDA), which yielded 82% sensitivity and 51% specificity, respectively (Blake et al., 2023). In contrast, Peng et al. (2023) reported that the random forest (RF) model provided superior diagnostic accuracy relative to PCA-LDA. After calibration with internal standard (IS) molecules, diagnostic accuracy exceeding 90% and specificity reaching 100% can be attained using decision tree (DT), RF, and PCA-LDA algorithms for differentiating cancerous from normal groups, and the calibrated AUCs were 0.9754, 0.996, and 0.9584, respectively (Peng et al., 2023). Overall, serum-based surface-enhanced Raman spectroscopy (SERS) profiles, combined with various algorithms, demonstrate potential for diagnosing normal and cancer groups, with the RF model showing higher diagnostic accuracy while PCA-LDA exhibits a weaker ability to identify colon cancer.

In summary, the integration of internal standard-calibrated SERS serum analysis with multivariate statistical algorithms is anticipated to serve as a highly precise and convenient liquid biopsy technique for non-invasive screening of colorectal cancer in clinical settings.

## 4 Applications of artificial intelligence-assisted Raman spectroscopy in diagnosis of head and neck cancer

Head and neck cancer constitutes a major global health burden, with an estimated 890,000 new cases and over 450,000 fatalities annually (Johnson et al., 2023). It is essential to develop more convenient and minimally invasive diagnostic approaches to improve early detection rates (Ghanem et al., 2025; Vlastou et al., 2025). Yang and colleagues reviewed the application of SERS in diagnosing head and neck cancer and found that SERS has attracted significant attention for its role in both diagnosis and treatment. This technique addresses critical challenges, including early detection and real-time intraoperative margin assessment (Yang B. et al., 2025). Furthermore, the synergistic integration of SERS with multi-omics approaches—such as genomics, proteomics, and metabolomics-holds the potential to revolutionize the understanding and management of head and neck cancer (Lima et al., 2021; Wu et al., 2023). Moreover, advancements supported by artificial intelligence (AI) are not only enhancing the quality of histopathological analysis but also accelerating it, promising to transform this centuries-old practice into a more efficient and insightful procedure that meets the demands of modern medicine (Abraham and Levenson, 2024). Consequently, the integration of SERS with cutting-edge technologies, including machine learning and deep learning algorithms that improve spectral analysis, heralds the dawn of a new era in precision oncology.

### 4.1 Nasopharyngeal carcinoma

Nasopharyngeal carcinoma (NPC) ranks as the eighth most common cancer among males in Singapore and is endemic in Southern China and Southeast Asia, exhibiting an incidence rate of 30–50 cases per 100,000 individuals (Chen et al., 2019). In 2023, Shu et al. identified essential biomolecules, such as chondroitin sulfate, glucose, hemoglobin, oleic acid, and triolein, from the Raman spectra of NPC tissues. Their study involved biomolecular modeling comparing early-stage NPC (stages I and II) and late-stage NPC patients (stages III and IV) (Shu et al., 2023). Furthermore, Shu et al. demonstrated the robustness of the RS-CNN model, which was developed to enhance *in vivo* Raman diagnosis of NPC and to facilitate rapid assessment of post-treatment efficacy for NPC patients during endoscopy. The high diagnostic sensitivity (92.18%) and specificity (73.99%) achieved in their work affirm the diagnostic effectiveness and clinical value of the deep learning-based Raman diagnostic platform for improving NPC detection and the rapid follow-up assessment of post-treatment outcomes in NPC patients (Shu et al., 2021). Xu et al. illustrated a metabolic map of seven NPC cell lines and successfully identified NPC and non-NPC cells using a single-cell Raman platform supported by various machine learning models, achieving high accuracy in classifying both cancer cells and patient tissues. An ROC-AUC of 0.99 was reported for classifying NPC and non-NPC cells and 0.97 was reported for classifying NPC tissues from nasopharyngitis tissues. Their data pave the way for a simple, less invasive, and accurate diagnostic test (Xu et al., 2024).

### 4.2 Oral squamous cell carcinoma

In 2020, the number of new cases of oral and lip tumors was staggering, reaching 377,713, with deaths from these tumors totaling 177,757 (Sung et al., 2021). Oral squamous cell carcinoma accounts for more than 90% of all oral cancer cases (Dumitrescu et al., 2025; D’souza and Addepalli, 2018). Two recent studies have investigated the application of AI-assisted RS for diagnosing oral squamous cell carcinoma (Li X. et al., 2023; Li et al., 2024). By leveraging fiberoptic RS alongside machine learning algorithms, Li et al. developed a single pathological diagnosis model that simultaneously performs multi-task network (MTN) diagnosis for both oral cancer pathological staging and histological grading. This model achieved accuracy rates of 94.88%, 94.57%, and 94.34% for tumor staging, lymph node staging, and histological grading, respectively (Li X. et al., 2023). The other study compared these multitask models with single-task models and traditional machine learning methods. Preliminary experimental results indicate that the multi-task network model performs well, with the MTN-Transformer achieving the best results. Specifically, the MTN-Transformer demonstrated an accuracy of 81.5%, precision of 82.1%, sensitivity of 80.2%, and an F1 score of 81.1% for tumor staging (Li et al., 2024). These findings underscore the potential value of AI-assisted RS in diagnosing oral squamous cell carcinoma.

## 5 Applications of artificial intelligence-assisted Raman spectroscopy in diagnosis of endocrine-related cancers

Endocrine-related cancers, typically referred to as sex steroid-responsive cancers, include breast cancer, endometrial cancer, prostate cancer, and testicular cancer, and encompass thyroid and ovarian cancers. These malignancies frequently disrupt hormone production, leading to various hormonal disorders (Xu et al., 2022; Miki et al., 2024). Growth factors, hormones, and their receptors serve as effective targets for precise diagnosis and therapeutic intervention for endocrine-related cancers. For instance, estrogen biosynthesis, estrogen receptors, and HER2 are relevant in breast cancer (Ventura et al., 2025; Yan et al., 2024), while androgen receptors are significant in prostate cancer (Danielli et al., 2025). Given the intricate molecular control networks associated with these cancers, RS serves as a non-destructive analytical technique that can rapidly deliver highly specific information regarding the biochemical composition and molecular structure of samples, making it well-suited for the study of endocrine-related cancers (Hanna et al., 2022; Ma et al., 2024; van Breugel et al., 2023). This section provides a summary of current published studies focusing on applications of AI-assisted RS in the diagnosis of thyroid cancer, breast cancer, and prostate cancer.

### 5.1 Thyroid cancer

Thyroid cancer is among the most prevalent endocrine malignancies, with its incidence rapidly increasing worldwide (Jaume, 2025). Early and accurate diagnosis of thyroid cancer is critical for effective treatment and improved patient outcomes, rendering it a significant public health issue (Jara and Castroneves, 2025). Sbroscia et al. demonstrated that Raman spectroscopy (RS) investigation of thyroid tissues provides reliable cancer diagnoses, achieving an accuracy of 90%. More importantly, Raman investigations have revealed alterations indicative of the early transition of adenoma tissues into cancerous tissues (Sbroscia et al., 2020). The spectral differences that distinguish benign and thyroid cancer cell lines were attributed to variations in the composition of nucleic acids, lipids, carbohydrates, and proteins. Acceptable sensitivities (74%–85%), specificities (65%–93%), and diagnostic accuracies (71%–88%) were achieved for identifying thyroid cancer (O'Dea et al., 2019).

With AI assistance, enhanced diagnostic performance for thyroid cancer has been attained (Song et al., 2024; Gao et al., 2024). Song et al. developed a novel multi-modal separation cross-fusion network (MSCNet) based on deep learning technology, which fully captures complementary information both between and within modalities through the feature separation and feature cross-fusion modules. This system effectively integrates Raman spectrum and Fourier-transform infrared (FTIR) spectrum data to accurately diagnose cervical lymph node metastasis in thyroid cancer. The analysis of 99 cases of cervical lymph node metastasis revealed that the accuracies for a single Raman spectrum and a single FTIR spectrum were 63.63% and 95.84%, respectively. After applying the feature separation and cross-fusion modules at the same time, MSCNet’s performance reaches the best, with an accuracy of 97.95% and the area under the curve (AUC) value of ∼98.00% (Song et al., 2024). Furthermore, Gao et al. developed a label-free SERS liquid biopsy method utilizing machine learning for the rapid and accurate diagnosis of thyroid cancer using thyroid fine-needle aspiration (FNA) washout fluids. Their findings indicate that the convolutional neural network (CNN) algorithm is the most precise, achieving an accuracy of 88.1%, a sensitivity of 87.8%, and an AUC value of ∼95.3% (Gao et al., 2024). This suggests that label-free SERS liquid biopsy, supported by deep learning models, holds substantial promise for the early detection and screening of thyroid cancer (Gao et al., 2024).

### 5.2 Breast cancer

Breast cancer is the most common cancer among women worldwide, with a 5-year survival rate of about 90% (Miller et al., 2022). However, due to the delayed treatments, breast cancer survivors suffer physical, functional and psychological sequelae that negatively affects their life. Therefore, establishing a straightforward, rapid, and efficient diagnostic approach is paramount for the treatment and monitoring of breast cancer prognosis (Kaur et al., 2024; Mathur et al., 2024). In recent years, the continuous advancement of RS and FTIR spectroscopy has found widespread application in the biomedical field for the diagnosis of breast cancer (Andras et al., 2025; Zhang S. et al., 2023). Deep learning techniques are often combined with single detection methods in this area to enhance the diagnostic accuracy, specificity, and sensitivity for breast cancer (Talari et al., 2019). This integration will be discussed in greater detail in the following subsections.

Shang et al. applied GoogLeNet to the fluorescence images, and obtained the discriminant accuracy of 89.5% and 88.61% for the validation sets and test sets. The AUC value was calculated as 0.9708, which confirms the satisfied discriminant ability of the trained GoogLeNet (Shang et al., 2020). These findings demonstrate that deep learning algorithms can be effectively applied to multiple diagnostic optics and spectroscopy techniques simultaneously, enhancing the accuracy of breast cancer diagnosis (Shang et al., 2020). Kothari and colleagues integrated laser Raman spectroscopy (LRS) with two machine learning algorithms—unsupervised k-means and stochastic nonlinear neural networks (NN). This combination achieved an accuracy of 93.2%–94.6%, a sensitivity of 89.8%–91.8%, and a specificity of 100%, facilitating rapid, quantitative, and probabilistic tumor assessment with real-time error analysis. Unsupervised k-means clustering and NN probability generation predict the likelihood of tumor for a larger dataset (n = 203, eight patients) (Kothari et al., 2021). However, they noted that machine learning algorithms utilized over the past decade in LRS breast cancer studies have often failed to provide two critical pieces of information essential for practicing surgeons: the probability that a classification is correct and the expected error associated with that probability. In contrast, stochastic backpropagation artificial neural networks inherently supply both of these pieces of information for individual tissue sites examined by LRS, rather than merely for clusters of data (Kothari et al., 2020; Nur Akkilic et al., 2023).

Cheng and coworkers employed SERS in combination with a two-dimensional convolutional neural network (2D-CNN) and Gramian angular field to analyze the serum of patients with breast cancer. Their findings revealed that the 2D-CNN-GAF method achieved an accuracy of 98.13%, a sensitivity of 98.65%, and a specificity of 97.67% for breast cancer classification. The AUC value of the 2D-CNN model (0.9884) is much higher than the AUC values of other algorithmic models (0.9704 for 1D-CNN, 0.9648 for KNN, 0.9613 for SVM and 0.9544 for PCA-LDA) (Cheng et al., 2024). Zhang et al. innovatively applied three distinct machine learning techniques—Random Forest (RF), Support Vector Machine (SVM), and CNN-alongside RS to streamline and expedite the differentiation between normal and late-stage cancerous mammary tissues in mice. The classification accuracy rates for these models were 94.47% for RF, 96.76% for SVM, and 97.58% for CNN, respectively. In addition, the CNN model achieved an unparalleled specificity and sensitivity of 99.51% and 95.65%. Its AUC value was 0.9842 (Zhang et al., 2024b). In the same year, Abramczyk’s research team developed an innovative methodology for HER2 protein identification in breast cancer cells by combining RS, Raman Imaging, and AI models, offering significant advantages over currently employed diagnostic methodologies (Abramczyk et al., 2024). In summary, advanced AI algorithms can enhance RS to improve the diagnostic accuracy, specificity, and sensitivity for breast cancer.

### 5.3 Prostate cancer

Prostate cancer is the most prevalent cancer among men globally, and its incidence in China has increased significantly in recent years (Wu et al., 2024). Unfortunately, current diagnostic and treatment methods for prostate cancer are constrained by a lack of accurate *in vivo* tissue analysis techniques (Picot et al., 2022; Centenera et al., 2022). In hindsight, applications of RS in prostate cancer include biopsy analysis, assessment of surgical margins and monitoring treatment efficacy (Gaba et al., 2022; Milligan et al., 2022). Among the various spectroanalytical techniques, SERS has been established as a promising method that significantly enhances Raman sensitivity when target biomolecules interact with a nanostructured surface, thereby providing reliable results for prostate cancer quantification (Haroon et al., 2022).

Shao et al. collected 1,281 Raman spectra from serum samples of 427 patients with prostate cancer to identify patients with bone metastases via label-free SERS and a CNN based on LeNet-5. Their study reported a training accuracy of 99.51%, a testing accuracy of 81.70%, a testing sensitivity of 80.63%, and a testing specificity of 82.82% (Shao et al., 2020). Subsequently, Wang and colleagues obtained SERS data from the serum of 729 patients diagnosed with either prostate cancer or benign prostatic hyperplasia and implemented an AI-assisted diagnostic model based on CNN, achieving an overall accuracy of 85.14% and an AUC value of 0.87 (Wang et al., 2023). Ge et al. applied a deep learning technique known as a multi-layer perceptron (MLP) to streamline the pre-processing of blood plasma SERS samples from patients with prostate cancer, enhancing both the sensitivity and specificity of the diagnosis using SERS technology. The classification accuracies for raw data, de-fluoresced data, and normalized data were 92.00%, 92.40%, and 96.70%, respectively (Ge et al., 2024). In conclusion, the integration of SERS analysis with various AI models holds significant potential for aiding the diagnosis of prostate cancer. Even so, larger datasets will further improve the models for rapid and automated prostate cancer screening in the future.

## 6 Applications of artificial intelligence-aided Raman spectroscopy in diagnosis of cervical cancer

Cervical cancer is the fourth most prevalent cancer globally, accounting for approximately 6.5% of all malignancies in women. The incidence and mortality rates of cervical cancer among women in China are notably high (Jha et al., 2023; Welby et al., 2022). Liu et al. combined coherent anti-Stokes RS (CARS) with a ConvNeXt network model based on CARS images to classify various types of tissue images, achieving a verification accuracy of 100% and a loss function of 0.0927 (Liu et al., 2025). Kang et al. conducted early screening for cervical cancer using tissue RS integrated with deep learning algorithms, discovering that the Efficient Channel Attention Convolutional Neural Network (ECACNN) exhibited the highest discrimination capability, with an average accuracy of 94.04%, F1 of 94.28% and AUC of 96.89% (Kang et al., 2023a). In the same year, the same research team employed H-CNN combined with tissue RS for cervical cancer detection, revealing that the classification Macro-Accuracy of H-CNN reached 94.91% (Kang et al., 2023b). Furthermore, a novel method based on RS, known as the Multi-level SENet Attention Mechanism Feature Fusion Architecture (MAFA), was proposed for the rapid diagnosis of cervical cancer and precancerous lesions. MAFA significantly enhances the diagnostic accuracy of VGGNet, GoogLeNet, and ResNet models in the validation of Raman spectral data from cervical tissue (Liu et al., 2023). In brief, these findings indicate that diagnostic models utilizing various AI algorithms can efficiently diagnose cervical cancer, providing new insights into the pathological diagnosis of this disease.

## 7 Applications of artificial intelligence-aided Raman spectroscopy in diagnosis of non-cancer diseases

In the final chapter of this review, we provide a succinct overview of preliminary studies investigating the role of AI-aided RS in diagnosing non-cancer diseases, including fungal infections and skin inflammation, as summarized in Table 2. While our primary focus remains on cancers, this discussion highlights the potential applications of AI and RS beyond oncological contexts.

Integrating AI with RS into routine clinical microbiology laboratory procedures has become increasingly intriguing. This integration promises to reduce turnaround times and costs while maximizing efficiency (Gao and Liu, 2024; Liu Y. et al., 2024; Dos Santos et al., 2023). Currently, at least one billion people are affected by fungal infections, resulting in over 1.6 million deaths annually (Denning, 2024; Epelbaum et al., 2025). Xu et al. constructed a Raman dataset from clinical fungal isolates obtained from 94 patients. By training a classification model with an optimized clinical feedback loop, this protocol achieved 100% accuracy in species-level fungal identification. Furthermore, this protocol was adapted for assessing clinical samples from urinary tract infections, allowing for accurate diagnoses from raw samples to results within 1 hour (Xu et al., 2023). Virus coinfections, where an individual is simultaneously infected by two or more pathogens, can markedly increase the severity of illnesses, complicate treatment, and lead to poorer health outcomes (Wang J. et al., 2025). Yang et al. developed a label-free diagnostic platform that integrates Surface-Enhanced Raman Spectroscopy (SERS) with deep learning for the rapid, quantitative detection of respiratory virus coinfections. This platform achieves an impressive 98.6% accuracy in classifying virus coinfections and a mean absolute error of 0.028 for concentration regression (Yang Y. et al., 2025). Such versatility enhances the platform’s potential as a rapid, point-of-care diagnostic tool with broad applications, promising a transformative impact in fields that require high sensitivity and specificity for diverse analytes and coexisting target mixtures.

In addition to infections, integration of AI and RS has also been utilized in the study of inflammatory diseases, including atherosclerosis and skin inflammation (Kanemura et al., 2022; Lee et al., 2023). Kanemura et al. combined near-infrared RS with AI analysis in a murine model to assess skin inflammation, employing a multilayer perceptron algorithm. Typical changes in the Raman spectra were observed during skin inflammation, which may have resulted from vasodilation and interstitial edema. Notably, the AI analysis improved the accuracy rate to 93.1% (Kanemura et al., 2022). Lee and colleagues proposed a novel method for diagnosing atherosclerosis in the carotid artery by utilizing nanometer biomarker measurements through label-free SERS from single-drop blood samples of *Apoe* ^*−/−*^ mice. Their results indicated that the principal component analysis-partial least squares regression-discriminant analysis (PCA-PLS-DA) machine learning algorithm achieved the highest accuracy of 97.5% (Lee et al., 2023). Collectively, these findings validate the considerable potential of integrating AI and RS in the diagnosis of inflammatory diseases.

In this context, additional diseases, including celiac disease, membranous nephropathy, and radiation-induced lung injury, have been reviewed. Celiac disease is an autoimmune disorder of the digestive system characterized by impaired fat digestion or absorption, resulting in the excretion of substantial amounts of fat and giving stools a milky appearance (Patel and Robert, 2022). Shi et al. utilized RS combined with deep learning models to develop a non-invasive, rapid, and accurate diagnostic method for distinguishing between celiac disease patients and healthy controls. Notably, they employed four types of classification models, including CNN, multi-scale convolutional neural network (MCNN), residual network (ResNet), and deep residual shrinkage network (DRSN). The results demonstrated that the DRSN model exhibited the best performance, achieving an accuracy of 95% (Shi et al., 2024). The second disease discussed is membranous nephropathy, the primary cause of nephrotic syndrome, which can have an insidious onset and may progress to end-stage renal disease, associated with a high mortality rate (Bose et al., 2022). Zhang et al. combined Raman spectra of serum and urine with three deep learning methods to diagnose membranous nephropathy, achieving a perfect accuracy of 1.0 for classifying serum data from patients with membranous nephropathy (Zhang X. et al., 2023). The last disease mentioned in this paragraph is radiation-induced lung injury, which poses a significant barrier to successful radiation therapy (Wang S. et al., 2025). Wiebe et al. employed RS and supervised machine learning to investigate metabolic associations with radiation pneumonitis and pulmonary fibrosis in a mouse model. They observed that group and basis-restricted non-negative matrix factorization classified with random forest (GBR-NMF-RF) was comparable to other methods in terms of accuracy and log-loss (Wiebe et al., 2024).

## 8 Concluding remarks

Raman spectroscopy (RS) is an emerging analytical technique that probes the molecular signatures of endogenous cellular biomolecules in biocompatible conditions while providing high spatial resolution. This technology has the potential to transform cancer diagnosis and immunotherapy by offering a non-invasive, high-throughput method for detecting molecular signatures in biofluids and tissue specimens. When combined with machine learning for real-time data analysis, these techniques position Raman technology as a disruptive tool throughout the continuum of oncological interventions. Although artificial intelligence (AI) has substantially advanced cancer diagnosis and management, it is limited by several issues that affect its reliability. For example, AI algorithms are highly dependent on the datasets used for training. If these datasets are biased or unrepresentative of ethnically diverse patient populations, the resulting AI models may experience overfitting, leading to inaccurate generalizations. Meanwhile, the interpretability of its decision-making process affects the accuracy. In addition, there are the core challenges for its clinical translation. Overall, the Raman technique, combined with intelligent algorithms, can be utilized for diagnosing liver and other types of tumors, potentially playing a significant role in pathological identification and intraoperative guidance. After extensive training with large datasets, AI models may assist RS in accurately and efficiently diagnosing prostate cancer.
